# The Focus of the Media Is Medical Intervention, Not the Pursuit of Health

**DOI:** 10.1371/journal.pmed.0020362

**Published:** 2005-10-25

**Authors:** Desmond A Gale

**Affiliations:** **1**Barbados Cancer SocietySt. MichaelBarbados

As the title of my response to the PLoS Medicine Debate [1] implies, the media know little and care less about the pursuit of health or about the requirements for health promotion, health maintenance, health protection, and disease prevention. Equally regrettable is the fact that very few journalists have the medical or scientific knowledge to qualify them to analyse and to think and write critically about medical policies and practices. Another shortcoming of the media is that their priority is not to educate and inform but to entertain the public. The outcome of this priority is that the most the public can expect from the media are meaningless fragments of information often calculated to confuse rather than enlighten them.
